# The Precision of Colour Doppler Ultrasonography Combined with Dynamic Infrared Thermography in Perforator Mapping for Deep Inferior Epigastric Perforator Flap Breast Reconstruction

**DOI:** 10.3390/jpm14090969

**Published:** 2024-09-13

**Authors:** Alex Victor Orădan, Alexandru Valentin Georgescu, Andrei Nicolae Jolobai, Gina Iulia Pașca, Alma Andreea Corpodean, Teodora Paula Juncan, Alexandru Ilie-Ene, Maximilian Vlad Muntean

**Affiliations:** 1 Department of Surgery—Plastic and Reconstructive Surgery, “Iuliu Hațieganu” University of Medicine and Pharmacy, 400012 Cluj-Napoca, Romania; oradan.alex@umfcluj.ro (A.V.O.);; 2 Department of Plastic and Reconstructive Surgery, Clinical Rehabilitation Hospital, 400066 Cluj-Napoca, Romania; 3 Department of Plastic and Reconstructive Surgery, “Prof. Dr. I. Chiricuță” Institute of Oncology, 400015 Cluj-Napoca, Romania; 4 Department of Diagnostic Radiology, “Prof. Dr. I. Chiricuță” Institute of Oncology, 400015 Cluj-Napoca, Romania; 5 Department of Plastic and Reconstructive Surgery, First Surgical Clinic, Emergency County Hospital, 400006 Cluj-Napoca, Romania; 6 Department of Surgery, “Iuliu Hațieganu” University of Medicine and Pharmacy, 400012 Cluj-Napoca, Romania

**Keywords:** perforator mapping, ultrasonography, thermography, breast reconstruction, DIEP flap, free flap

## Abstract

**Background**: Perforator mapping is a mandatory tool for the preoperative planning of a microsurgical free flap, especially in breast reconstruction. Numerous methods for mapping have been described. In this study, we investigate the combined use of Dynamic Infrared Thermography (DIRT) and Colour Doppler Ultrasonography (CDUS) only to see whether it can eliminate the need for Computed Tomography Angiography (CTA). **Methods**: A prospective study was conducted on 33 patients with deep inferior epigastric perforator (DIEP) flaps for breast reconstruction. DIRT, followed by CDUS and CTA, was performed preoperatively and perforators were confirmed intraoperatively. **Results**: From 135 hot spots found on DIRT, 123 perforators were confirmed by CDUS (91.11%). A total of 86.66% of the perforator vessels detected on CTA have their correspondent on DIRT, while 95.12% have their correspondent on CDUS. No statistically significant difference (*p* > 0.05) was found comparing DIRT vs. CTA and CDU vs. CTA. The average DIRT time was 121.54 s and CDUS 232.09 s. The mean sensitivity for DIRT was 95.72% and 93.16% for CDUS. **Conclusion**: DIRT combined with CDUS can precisely and efficiently identify suitable perforators without the need for CTA in DIEP breast reconstruction.

## 1. Introduction

Breast cancer is the most common type of cancer in women worldwide and has a big impact on the patient’s quality of life [1,2,3]. Immediate or delayed breast reconstruction following mastectomy has become a crucial part of the modern treatment of breast cancer and with the help of new advances in surgery, it is able to restore the natural symmetry, and aesthetic looks, and also improve self-confidence [4,5,6]. Microsurgical breast reconstruction using redundant abdominal tissue was introduced by numerous authors around three decades ago and has become popular in the modern era of reconstructive surgery by means of the deep inferior epigastric perforator (DIEP) flap [7,8]. It is widely accepted that the DIEP flap was first described by Koshima and Soeda in 1989 [9]. Since its introduction, it has grown to become the first choice in autologous breast reconstruction with superior aesthetic results and low morbidity rates [10]. The survival of a perforator flap relies on the number of perforators and their position within the flap to enable good tissue perfusion [11]. A thorough understanding of the anatomy and a concise preoperative mapping of the perforators are mandatory for DIEP flap planning. In this way, with reduced surgical time and improved flap safety, the postoperative result will be successful [6,11].

There are numerous methods used for perforator mapping such as hand-held Doppler (HHD), Colour Doppler Ultrasonography (CDUS), Computed Tomography Angiography (CTA), Magnetic Resonance Angiography (MRA), and Dynamic Infrared Thermography (DIRT), each with its advantages and disadvantages [12,13,14,15,16,17]. Based on the “Gent” consensus, CTA is considered the gold standard for perforator mapping [18]. Currently, it is considered the preferred method in DIEP reconstruction with a high sensitivity and positive predictive value which allows the precise localisation and selection of DIEA perforators compared with the operative findings [15,19,20]. However, CTA is associated with high costs, radiation, and contrast exposure [19,21].

CDUS is the most common technique used for the preoperative perforator location [22,23]. It is a dynamic method with an accurate description of the perforator vessels, with no radiation exposure, adding supplementary information about the diameter and velocity of perforators. Also, it is not considered inferior to CTA despite the unpredictability between pre- and intraoperative detection [21]. It can be a complementary or substitutive modality for CTA mapping, even with a portable device and conducted by the surgeon [24]. The main limitations are the high observer dependency and that it can be time-consuming [17]. 

The first medical description of infrared thermography (IRT) imaging was in 1956, when Lawson et al. observed that the skin temperature on normal breast parenchyma was lower than the skin above breast cancer [25]. This method was capable of detecting the distribution of “hot and cold spots” in the body area. From that point on, with the evolution of technology, it has become more available, and it can be used to easily monitor the thermal recovery after exposing a region to thermal stress. This revolutionary technique is called Dynamic Infrared Thermography (DIRT). The first interpretation of DIRT for DIEP mapping was performed in 1993 [26]. DIRT is a dynamic non-invasive technique which measures skin temperature based on the heat emitted by tissue. It detects the distribution of the so-called “hot spots” and is able to generate a colour-coded map which can be used to map perforators [12,27]. DIRT is an efficient, widely available method with low costs which can provide reliable information about skin perfusion, but still with limited evidence regarding its utility for perforator mapping pre- and intraoperatively [17]. Nevertheless, many confounding factors can interfere with the imaging attainment such as core body temperature, external temperature, and system calibration [16].

In this study, we analysed preoperative perforator mapping using CDUS combined with DIRT and CTA for breast reconstruction with autologous tissue with DIEP flaps. The purpose of this work is to evaluate the feasibility, precision, and ease of use by combining CDUS with DIRT without the need for CTA in order to accurately perform the mapping, to explore the effect of this preoperative step with the intraoperative findings, and to discuss the eventual benefits when using this combined method. 

## 2. Materials and Methods

This prospective study was conducted in the plastic surgery department of “Prof. Dr. Ion Chiricuta” Institute of Oncology, Cluj-Napoca, Romania. All the subjects gave their informed consent for inclusion before they participated in the study. The study was conducted in accordance with the Declaration of Helsinki and approved by the Institutional Review Board. The study spanned over a period of two years (from March 2021 until August 2023). The patients included were female patients who had previously undergone mastectomy and adjuvant treatment for breast cancer and were good candidates for delayed DIEP flap breast reconstruction (redundant abdominal tissue and non-smokers). The patients with known vascular anomalies, previous procedures on the lower abdomen, or other medical conditions causing hyperkinetic blood flow were excluded. 

Preoperatively, perforator mapping was performed by means of DIRT, CDUS, and CTA for each patient one day prior to the surgery. DIRT and CDUS were performed by the same plastic surgeon (AO) without knowing the results of the CTA, therefore blinding the examiner (AO). The first step in the perforator mapping was to mark the hotspots on the abdomen using DIRT and afterwards perform CDUS on each separate hotpot in order to locate a potentially suitable perforator. The CTA examination results were interpreted by a radiologist (GP). 

### 2.1. Imaging Examination

First, DIRT was performed using a thermal camera (Testo 890 Handheld Thermal Imaging Camera—Testo AG, Lenzkirch, Germany) with a thermal sensitivity of <40 mK and a standard lens. The abdomen was cooled by pulverising alcohol and delivering air at room temperature by the mean of a fan for 60 s. The area was continuously monitored with the thermal camera through the rewarming phase. Hot spots were identified on the infra-umbilical abdominal wall. The hot spots were traced on the skin with a marker and a point corresponding to the centre of the hot spot was marked. All the points were measured both laterally and inferiorly with the umbilicus as a reference point and the distances were recorded in centimetres. The total time for the DIRT examination was recorded (seconds).

Next, CDUS was performed (Samsung RS85—Samsung Medison CO., Ltd., Schwalbach am Taunus, Germany) with a linear 12 MHz transducer in B-mode and colour Doppler mode directly on the previously marked hot spots to identify possible perforating vessels from the deep epigastric artery. CDUS was performed solely on the previously marked hot spots; therefore, there was no point in searching for other perforators outside the hot spots. The total time for CDUS and the time required to identify the perforator for each hot spot was recorded (seconds). The point where the perforator emerged at the level of the fascia was marked on the skin and measured from the umbilicus laterally and inferiorly in a similar fashion to the DIRT markings and recorded.

CTA scans were obtained with a 64-slice Somatom Confidence (Siemens, Munich, Germany). The bolus trigger region of interest was at the common iliac artery; 100 mL iodinated contrast agent was injected at a rate of 3.5–4 mL/s using a 6 s delay for arterial-phase images at a 0.65 mm slice thickness. The scan was performed caudo-cranially, with the patient in a supine position with their arms lying upwards, from the pubis to 3 cm above the umbilicus using a 120 kV 250–350 mA, table pitch 1, zero gantry pitch protocol, with automatic tube current modulation and 0.6 mm collimation width. The images are reconstructed with a slice thickness of 1 mm. For data measurement on CTA, a diagnostic software was used to measure the coordinates of the perforators relevant to a DIEP flap on the CTA scans. These measurements were conducted similarly to the method described above using the umbilicus as the reference point. The data were then analysed to correlate the coordinates of the perforators identified during DIRT plus CDUS with those found on CTA. 

### 2.2. Intraoperative Findings

**Figure 1:**
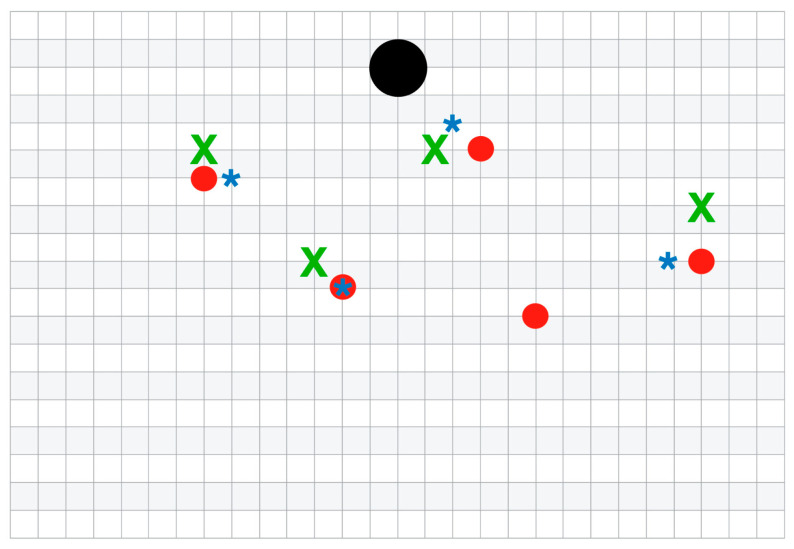
Preoperative mapping. After performing DIRT, CDUS, and CTA, the coordinates are placed on a grid with one square representing 1 cm^2^ by measuring the distance from the umbilicus (black circle). The centre of the hotspots is represented by a red circle. The blue asterisk (*) represents the fascial emergence of the perforator on CDUS. The green “X” represents the fascial emergence of the perforator on CTA.

Finally, after DIRT, CDUS, and CTA, the perforators were assessed intraoperatively and were selected for flap harvest based on the morphological findings and location. The same team that had performed the preoperative mapping conducted the breast reconstructive surgery. An image was printed with all the preoperative markings found with DIRT, CDUS, and CTA (Figure 1). Every time a suitable and sizeable perforator with visible pulsations came across during the flap harvest, it was marked on the printed image to note the equivalent with the preoperative one. Smaller perforators were divided. The number and location of the perforators identified by DIRT, CDU, and CTA were compared with the intraoperative findings.

### 2.3. Statistical Analysis

Database analysis was performed in Microsoft Excel (Microsoft Office Professional Plus 2021) and SPSS 27.0 (IBM, Armonk, NY, USA). The two types of continuous variables (the number of identified perforator vessels and examination time) are presented as mean +/− standard deviation. Differences between DIRT and CTA or CDU and CTA were estimated using a two-tailed *t*-test. A *p*-value < 0.05 was considered statistically significant and the confidence interval was 95%. 

Afterwards, the sensitivity of each technique was evaluated by comparing it to the intraoperative findings. A true positive value was allocated for each perforator marked initially with the 3 methods and corresponding to an accurate intraoperative finding. On the contrary, when the preoperative exam found a perforator without any correspondence during surgery, a false positive value was attributed. Since the condition of the absent perforators cannot be determined, there was no true negative value discovered. The specificity of the methods was not evaluated.

## 3. Results

A total of 33 unilateral DIEP breast reconstructions were performed. In total, 117 perforators were detected intraoperatively. Preoperatively, 135 hot spots were identified with DIRT, 123 with CDUS and 117 with CTA. Based on the 135 hot spots, 123 perforators were found using CDUS (91.11%). A total of 86.66% of the perforator vessels detected on CTA have their correspondent as a hot spot, while 95.12% have their correspondent on CDUS. No statistically significant difference (*p* > 0.05) was found comparing DIRT vs. CTA and CDUS vs. CTA. The sensitivity for each detection method was calculated and is listed in Table 1. The number of hot spots and perforators identified with each method is listed in Table 2.

The average DIRT time was 121.54 s (+/−40.41 SD) and the median DIRT time was 120 s. For CDUS, we achieved an average of 232.09 s (+/−65.16 SD) and a median of 238.5 s (61.78 s average time necessary for each individual perforator analysis). The mean difference between the two methods: 110.55 s, t = −2.34, *p* < 0.05.

## 4. Discussion

Perforator mapping is an important tool in the planning of a free flap, particularly in breast reconstruction where success has a high impact on the patient’s quality of life. CTA has long been considered the gold standard among other methods such as HHD, MRA, CDUS, and DIRT, especially in DIEP flaps. Nonetheless, we were able to prove that similar results can be obtained with DIRT and CDUS alone without the need for CTA, therefore eliminating the need for exposure to a costly and invasive examination procedure.

CTA provides information regarding the number, size, course, and location of the perforators preoperatively, but it does not provide hemodynamic information, is costly, and exposes the patient to radiation and intravenous contrast agents [19,21]. Moreover, it enables the possibility to perform 3D imaging and provide the branching patterns of the deep inferior epigastric artery and a global view of all the perforators [19,28]. Based on a consensus, CTA is considered the preferred method for perforator mapping, with better spatial resolution compared to MRI and more sensitivity than ultrasound [18,29]. According to the literature and our own findings, the sensitivity of CTA in perforator mapping reaches 100% [11,20,28,30,31,32], although one recent study reports a correlation of 70% of the clinically selected DIEPs identified on preoperative imaging (CTA and MRI) compared to the nearly 100% predictive value reported by others [33]. In our clinical practice, we currently perform CTA for every DIEP patient in order to precisely map the perforators, although we have observed that the accuracy of our DIRT and CDUS closely matches the results found on CTA; therefore, we believe that CTA is redundant.

The main advantages of CDUS are the precise localisation of perforator position cutaneously with additional parameters such as velocity and diameter and without radiation exposure [21,34]. The examination can be performed even by the surgeon with portable devices with good results [24,35]. Some do not recommend HHD due to its inability to accurately identify the perforator relative to the surrounding tissue [11]. Even though most studies emphasise the superiority of CTA over CDUS, Mijuskovic et al. reported superior results in the accuracy of the selection and number of detected perforators using CDUS compared to CTA [13,36,37,38]. Based on our findings, we believe that CTA continues to be superior to CDUS (highest sensitivity), although not always necessary for performing a successful DIEP reconstruction. In a recent study, CDUS detected significantly more perforators than CTA and a strong correlation between the dominant perforator in CDUS and the intraoperatively selected one [13]. A possible explanation is that in this study, only highly specialised angiologists performed the CDUS; therefore, it can be considered a reliable diagnostic tool without the risk of radiation. Contrast-Enhanced Ultrasound (CEUS) is a feasible supplement or may even function as an alternative to CTA according to a new study. It allows precise perforator mapping with a sensitivity of 91.2% and a specificity of 88.9% without concomitant radiation. The main disadvantage of this technique is that it requires an experienced sonographer [11,39]. CEUS average examination time was 45 min, while others report times up to two hours [11,40]. In our study, the mean time to perform CDUS after DIRT was around 3–4 min (232.09 s), significantly lower than the literature given the fact that it was not performed by an experienced sonographer.

DIRT uses an infrared camera to measure the skin temperature based on the heat emitted by tissues. This generates a colour-coded map, which is a translation for the perfusion of the skin [12,27]. In clinical practice, DIRT is useful for mapping perforators, optimising flap design, assessing blood flow, and evaluating anastomosis patency [14]. It was first used for perforator mapping in DIEP in 1993 [26]. Smartphone-enabled thermal cameras are readily available currently and have proven to be useful and accurate, while artificial intelligence can help the surgeon select the most suitable perforators [41,42,43,44,45]. The flow in perforator vessels emits a detectable heat signal, which can be recognised by infrared thermography thus allowing localisation [44]. DIRT is a dynamic investigation technique because the skin surface is first cooled and allowed to rewarm in order to observe the pattern of rewarming. After cooling the surface, the temperature difference is analysed during the rewarming phase. Cooling methods include applying a hemostatic clamp on the vessel, cold water packs, blowing cold air, and the extraction of heat by alcohol evaporation from the skin surface. A standardised cooling method with 3 min of cooling with 5 degrees water has been described [46]. It is inexpensive, portable, less invasive than CTA, with no radiation, and has no contraindications, which shows promising results for the preoperative localisation of perforator vessels [44]. According to a recent study, DIRT has a high resemblance to CDUS and CTA [27]. DIRT only provides information on the physiology of the perforator, not on the morphology, as brighter hot spots may be an indicator of perforator quality, offering additional information in the surgical decision-making process [16,44]. Moreover, similar to CTA, DIRT provides a global view of all the potential perforators in one area. In our case, the mapping procedure starts with DIRT in order to offer a global view and make the CDUS easier, more precise, and save time. Although we did not add a control group with CDUS only, in our previous animal study, the time required to successfully identify perforators was less using DIRT followed by CDUS rather than CDUS alone [12]; therefore, performing a so-called “on the (hot)spot” CDUS also shortens the examination time clinically (Table 1).

Regarding the accuracy of the examination, our previous study showed that DIRT can correctly identify the dominant perforator with a higher sensitivity than CDUS. Other papers have demonstrated similar results [12,46,47,48]. In one study by Weum et al., from 113 hot spots identified with DIRT, 108 had their correspondent on CTA (95.6%) [47]. Similarly, we obtained 86.66%. Compared to CDUS, DIRT has a lower positive predictive value and produces more false-positive results due to perforator branching at the skin level [12]. Our evidence suggests a lower sensitivity for CDUS compared to DIRT (Table 1). Although DIRT correlated well with CTA, but less with CDUS in a previous publication [14], we have found out that performing CDUS directly on the hot spot can correctly identify a perforator (91.11% of the hotspots corresponded to a detectable perforator on CDUS). Nevertheless, CTA findings still match the ones on DIRT and CDUS.

Finally, the limitations of this study are the small number of subjects included, and future studies on larger cohorts are needed to support our evidence with a more accurate localisation and correlation of perforators for each examination by the region of interest. Furthermore, another limitation is the fact that only the potential perforators found using DIRT were assessed by CDUS; therefore, the sensitivity of CDUS can only be as high as the sensitivity of DIRT.

## 5. Conclusions

We conclude that performing CDUS in conjunction with DIRT in an “on the (hot)spot” manner allows the surgeon to precisely and efficiently map perforators suitable for DIEP breast reconstruction even without CTA. Since this study provides promising results, further research should focus on increasing the sensitivity of DIRT and CDUS, which may become a promising replacement for a costly, time-consuming, and invasive CTA. 

## Figures and Tables

**Table 1:** Comparison of the imaging techniques.

Features	DIRT	CDUS	CTA
Invasiveness	None	None	Intravenous contrast
Radiation exposure	None	None	Ionising radiation
Sensitivity	95.72%	93.16%	100%
Time needed for a complete examination	2–3 min	3–4 min	Hours
Ease of use	Yes	Yes	No

**Table 2:** The number of findings for each patient by the means of DIRT, CDUS, CTA, and intraoperatively.

Patient	Hot Spots Identified with DIRT	Perforators Identified with CDUS	Perforators Identified with CTA	Suitable Perforator Encountered Intraoperatively
Patient no. 1	5	4	5	5
Patient no. 2	5	4	4	4
Patient no. 3	4	4	4	4
Patient no. 4	3	3	3	3
Patient no. 5	4	4	3	3
Patient no. 6	3	3	3	3
Patient no. 7	3	3	3	3
Patient no. 8	5	5	4	4
Patient no. 9	4	4	3	3
Patient no. 10	5	4	4	4
Patient no. 11	4	3	3	3
Patient no. 12	3	3	3	3
Patient no. 13	5	5	4	4
Patient no. 14	4	4	3	3
Patient no. 15	5	4	4	4
Patient no. 16	4	3	3	3
Patient no. 17	3	3	3	3
Patient no. 18	3	3	3	3
Patient no. 19	5	4	4	4
Patient no. 20	5	4	5	5
Patient no. 21	4	4	4	4
Patient no. 22	4	4	3	3
Patient no. 23	4	5	4	4
Patient no. 24	4	4	3	3
Patient no. 25	3	3	2	2
Patient no. 26	5	4	5	5
Patient no. 27	3	3	2	2
Patient no. 28	5	4	4	4
Patient no. 29	4	4	4	4
Patient no. 30	4	3	3	3
Patient no. 31	4	4	4	4
Patient no. 32	4	4	4	4
Patient no. 33	5	3	4	4

## Data Availability

The raw data supporting the conclusions of this article will be made available by the authors upon request.
